# Short-Step Adjustment and Proximal Compensatory Strategies Adopted by Stroke Survivors With Knee Extensor Spasticity for Obstacle Crossing

**DOI:** 10.3389/fbioe.2020.00939

**Published:** 2020-08-06

**Authors:** Shang-Jun Huang, Xiao-Ming Yu, Kuan Wang, Le-Jun Wang, Xu-Bo Wu, Xie Wu, Wen-Xin Niu

**Affiliations:** ^1^Key Laboratory of Spine and Spinal Cord Injury Repair and Regeneration of Ministry of Education, Orthopaedic Department, Tongji Hospital, Tongji University School of Medicine, Shanghai, China; ^2^Department of Rehabilitation, Shanghai Seventh People’s Hospital, Shanghai University of Traditional Chinese Medicine, Shanghai, China; ^3^Yangzhi Rehabilitation Hospital, Tongji University School of Medicine, Shanghai, China; ^4^Sport and Health Research Center, Physical Education Department, Tongji University, Shanghai, China; ^5^School of Rehabilitation Medicine, Shanghai University of Traditional Chinese Medicine, Shanghai, China; ^6^Key Laboratory of Exercise and Health Sciences, Ministry of Education, Shanghai University of Sport, Shanghai, China

**Keywords:** stroke, spasticity, compensatory strategy, gait, biomechanics

## Abstract

Stroke survivors adopt cautious or compensatory strategies for safe and successful obstacle crossing. Although knee extensor spasticity is a common independent secondary sensorimotor disorder post-stroke, few studies have examined the step adjustment and compensatory strategies used by stroke survivors with knee extensor spasticity during obstacle crossing. This study aimed to compare the differences in the kinematics and kinetics during obstacle crossing between stroke survivors with and without knee extensor spasticity, and to identify knee extensor spasticity-related differences in step adjustment and compensatory strategies. Twenty stroke subjects were divided into a spasticity group [*n* = 11, modified Ashworth scale (MAS) ≥ 1] and a non-spasticity group (*n* = 9, MAS = 0), based on the MAS score of the knee extensor. Subjects were instructed to walk at a self-selected speed on a 10-m walkway and step over a 15 cm obstacle. A ten-camera 3D motion analysis system and two force plates were used to collect the kinematic and kinetic data. During the pre-obstacle phase, stroke survivors with knee extensor spasticity adopted a short-step strategy to approach the obstacle, while the subjects without spasticity used long-step strategy. During the affected limb swing phase, the spasticity group exhibited increased values that were significantly higher than those seen in the non-spasticity group for the following measurements: pelvic lateral tilt angle, trunk lateral tilt angle, medio-lateral distance between the ankle and ipsilateral hip joint, hip work contributions, the inclination angles between center of mass and center of pressure in anterior–posterior and medio-lateral directions. These results indicate that the combined movement of the pelvic, trunk lateral tilt, and hip abduction is an important compensatory strategy for successful obstacle crossing, but it sacrifices some balance in the sideways direction. During the post-obstacle phase, short-step and increase step width strategy were adopted to reestablish the walking pattern and balance control. These results reveal the step adjustment and compensatory strategies for obstacle crossing and also provide insight into the design of rehabilitation interventions for fall prevention in stroke survivors with knee extensor spasticity.

## Introduction

Stroke often results in spasticity and associated motor impairments in the lower limbs, including muscle weakness (Li et al., 2014), proprioceptive deficit (Gorst et al., 2019), abnormal agonist-antagonist coactivation (Trumbower et al., 2010), and altered inter-joint and inter-segmental coordination (Subramanian et al., 2018; Salehi et al., 2020). In clinical rehabilitation, the spasticity and movement deficits disorders have been traditionally been considered as separate phenomena (Levin, 2016). However, spasticity is not just an independent disorder, it has a negative effect on other motor disorders. One previous study demonstrated that spasticity affects passive tissue stiffness and disrupts the agonist-antagonist activation pattern, which alters the net effect of the forces generated by the muscle groups (Singer et al., 2013). In addition, lower limb spasticity can interfere with proprioceptive inputs, inter-limb coordination, and balance control in stroke survivors (Singer et al., 2013; Singer and Mochizuki, 2015; Yang and Kim, 2015; Rahimzadeh Khiabani et al., 2017). As a result, community ambulation tasks, such as obstacle crossing, can be more challenging for stroke survivors with lower limb spasticity than for those without (Soyuer and Ozturk, 2007).

Obstacle crossing is a complex task in community ambulation (Raffegeau et al., 2019). When healthy adults step over obstacles, a ‘knee strategy’ is used, whereby mechanical energy is generated above the knee joint to flex both the knee and hip simultaneously (MacLellan et al., 2015). Obviously, the hip flexor (knee extensor) and knee flexor together are the main power sources for clearance and limb swing. Motor disorders after a stroke cause subjects to fall during obstacle crossing due to imbalance or tripping (Said et al., 2013; Ma et al., 2017; Punt et al., 2017). One basic motor skill of stroke survivors is the ability to initiate an appropriate movement strategy to counter the change in environmental conditions and task demands based on their functional level, including step adjustment and compensatory strategies to complete the task and maintain balance (Banina et al., 2017; Malik et al., 2017; Shafizadeh et al., 2019). Several such strategies for safe and successful obstacle crossing have been reported in stroke survivors, by comparing the obstacle crossing biomechanics of stroke survivors and age-matched healthy controls subjects, respectively (Said et al., 2008; Lu et al., 2010; Nakano et al., 2014; MacLellan et al., 2015; Chen et al., 2019; Shafizadeh et al., 2019). Experimental studies of these strategies may aid our understanding of motor control, and may help in the development of better fall prevention training programs.

During unperturbed human walking, the spatial and temporal characteristics of the steps are relatively fixed and typically show low step-to-step variability (Den Otter et al., 2005). Step modifications often involve rigorous re-parameterization of forces and require a high level of neuromuscular control (Sun et al., 2017). Consequently, stroke survivors with impaired neuromuscular functioning may be more vulnerable to perturbations of the motor task and ongoing stepping pattern (Den Otter et al., 2005). When stroke survivors approach an obstacle, the steps must be modified in due time to minimize disturbance to the gait, even in the absence of temporal constraints. Nakano et al. (2014) suggested that the short-step strategy was used by stroke survivors to approach the obstacle, which probably was intended to enhance the accuracy of swing and maintain stability. Additionally, several studies demonstrated that a “hip abduction strategy” was adopted by stroke survivors to compensate for the lack of knee flexion in crossing the obstacle successfully (Lu et al., 2010; Chen et al., 2019). Inconsistently, a previous study demonstrated that the “residual knee flexor and hip flexion strategy” is still present in the affected limb, and augmented by hip elevation and flexion (MacLellan et al., 2015). Although knee extensor spasticity is a common and independent secondary sensorimotor disorder post-stroke, few studies have examined the step adjustment and compensatory strategies used by stroke survivors with knee extensor spasticity during obstacle crossing.

Spatiotemporal parameters and joint kinematics provide information about adjustments and compensatory strategies for successful obstacle crossing and balance. One previous study reported that stroke survivors were more unstable than healthy adults (Said et al., 2005). To compensate for this instability, stroke survivors reduced the anterior–posterior (AP) speed of their center of mass (COM), shortened their step length, and shifted their COM posteriorly when the affected limb was crossing an obstacle. Chen et al. (2019) reported that the hip abduction angles and pelvic medio-lateral (ML) tilt angles of stroke survivors are larger than those of healthy subjects when the affected limbs are crossing obstacles. Additionally, recent studies have suggested that the COM-COP (center of pressure, COP) inclination angle could sensitively identify individuals with imbalance and fall risk among stroke survivors during walking and obstacle crossing (Chen and Chou, 2010; van Vugt et al., 2019). Hence, spatiotemporal parameters and joint kinematics were helpful in identifying the compensatory strategies and risk of falling during obstacle crossing.

Kinetic analysis provides insight into how to elevate lower limb to step an obstacle. As mentioned previously, the hip abduction strategy is an important compensatory strategy for successful obstacle crossing in stroke survivors. Inconsistently, MacLellan et al. (2015) studied the kinetic strategies of stroke survivors for obstacle avoidance with either the affected or unaffected as the leading limb, and demonstrated that the residual knee flexor strategy is still present in the paretic limb, and augmented by hip elevation and flexion. Furthermore, one previous study reported that estimating the relative contribution of each joint to the total energy generated during the swing phase is a useful approach for understanding the degree of compensation strategy (Teixeira-Salmela et al., 2008). Therefore, the joint work and work contributions can be used to examine compensatory strategies for successful obstacle crossing.

The purpose of this study was to systematically examine the step adjustment and compensatory strategies used by stroke survivors with knee extensor spasticity during obstacle crossing. To achieve this goal, we compared the biomechanics of obstacle crossing between stroke survivors with and without knee extensor spasticity. A previous study suggested that the coupling of movement between the pelvic and trunk contributed to the compensatory strategy for complex tasks (Malone et al., 2016; Han et al., 2017). Therefore, we hypothesized that stroke survivors with knee extensor spasticity rely more on the trunk movement compared to those without spasticity when the affected limb is crossing an obstacle. A previous study showed that the degree of trunk movement was restricted in order to enable body stability in the early stage of motor learning and balance development (Rhee and Kim, 2015). Due to the excessive movement of the trunk and pelvic, we also hypothesized that stroke survivors with knee extensor spasticity have a higher COM-COP ML inclination angle than those without spasticity.

## Materials and Methods

### Study Design

A biomechanical cross-sectional study was conducted to compare the differences in the kinematics and kinetics between stroke survivors with and without knee extensor spasticity during the crossing of 15 cm, and to identify the knee extensor spasticity-related changes in step adjustment and compensatory strategies for obstacle crossing.

### Sample Size

The sample size was calculated using G^∗^power software (v3.1.9.2) based on a comparison of the pelvic lateral tilt angle between the stroke survivors and age-matched healthy controls during obstacle crossing. A previous study showed that the means and standard deviations of the pelvic lateral tilt angle were 12.12° and 5.87°, and 8.14° and 9.38° in the stroke and healthy control groups, respectively (Chen et al., 2019). According to a prior one-way ANOVA *F*-test, with a power of 0.80 and an alpha level of 0.05, an estimated 14 participants were required for this study.

### Participants

Twenty stroke subjects were recruited from the Seventh People’s Hospital and community centers in the vicinity (Gaoqiao, Pudong District, Shanghai, China) using flyers, posters, and referrals from neurologists and physical therapists between October 2017 and November 2018. The basic characteristics of the subjects are shown in Table 1. The inclusion criteria were as follows: clinical diagnosis of cerebral hemorrhage or infarction by computed tomography/magnetic resonance imaging (CT/MRI), 30–75 years of age, ≥3 months since the stroke, a score of >24 on the Mini-Mental State Examination (MMSE), ability to walk 10 m without a gait aid or assistance, without the history of using ankle-foot orthoses, no botulinum toxin drug treatment within 3 months and ability to cross an obstacle with a height of 15 cm. The exclusion criteria included current involvement in any other clinical study or instructor-directed exercise program, vision disorders, severe hypertension or cardiopulmonary diseases, and lower limb joint or muscle injuries.

**TABLE 1:** Basic characteristics of study subjects.

Characteristics	Spasticity group	Non-spasticity group	*F*/χ^2^	*P*
Age (years)	60.02 (54.09–65.91)	61.33 (55.54–67.12)	0.129	0.724
Height (m)	1.72 (1.66–1.75)	1.69 (1.66–1.72)	0.799	0.383
Mass (kg)	73.81 (69.67–77.93)	67.39 (64.28–70.50)	4.316	0.052
Time post-stroke (months)	11.39 (7.44–15.34)	9.35 (4.54–14.16)	0.562	0.463
Sex (female/male)	9/2	6/3	0.617	0.396
Type of stroke (Isc/Hem)	9/2	5/4	0.336	0.217
Affected side (left/right)	6/5	4/5	0.000	1.000
MAS (score)	1.45 (1.14–1.77)	0.00 (0.00–0.00)	84.637	0.000
MMSE (score)	27.55 (26.19–28.90)	28.11 (26.17–30.05)	0.311	0.584
FMA (score)	25.82 (22.20–31.25)	28.44 (26.09–30.35)	1.201	0.288
BBS (score)	45.73 (45.51–51.40)	48.89 (47.75–51.59)	3.519	0.077

*A score of 1+ on the modified Ashworth scale was entered as 1.5. Isc, ischemic stroke; Hem, hemorrhagic stroke; MAS, modified Ashworth scale; MMSE, mini-mental state examination; FMA, Fugl-Meyer assessment; BBS, Berg Balance Scale.*

A modified Ashworth scale (MAS) was used to assess the resistance to passive movement and to indirectly measure spasticity level of the knee extensor (Ansari et al., 2008; Meseguer-Henarejos et al., 2018). The subjects were instructed to lie on a bed, with their hips and knees in extension. Behind the subject, the evaluator placed one hand just proximal to the knee to stabilize the femur, and the other hand grasped the leg just proximal to the ankle. The subject’s knee was flexed from a position of maximal possible extension to maximal possible flexion over a duration of approximately 1 s (Blackburn et al., 2002). Only one movement was allowed to determine the resistance to passive movement (Ansari et al., 2008). A single physical therapist with many years of assessment experience performed all evaluations, in order to eliminate extraneous variability in the assessment results and to ensure accuracy. Twenty stroke subjects were divided into two groups: subjects with spasticity (*n* = 11, MAS ≥ 1) and a control group of stroke subjects without spasticity (*n* = 9, MAS = 0), according to the MAS score of the knee extensor. This study was approved by the institutional review board of Shanghai Seventh People’s Hospital (2018-IRBQYYS-012). Informed consent was obtained from all participants enrolled in the study.

### Apparatus

A total of 50 spherical 14-mm infrared-reflective markers were fastened to each subject’s body according to the Vicon Plug-in-Gait model. A 10-camera 3D motion analysis system (Vicon Motion Systems, Oxford, United Kingdom) recorded the marker trajectory data at a sampling frequency of 100 Hz. Two force plates (900 mm × 600 mm × 140 mm, Kistler Instruments AG Corp., Switzerland) recorded the kinetic data at a sampling frequency of 1,000 Hz. The data from the 3D motion analysis system and force plates were systematically synchronized using the terminal box of an Analog/Digital converter. The obstacle consisted of two upright stands with a lightweight crossbar of adjustable height. The obstacle was placed in the middle of the two force plates. Two markers were attached to the obstacle to mark the relative position between the obstacle and the subject.

### Procedure

Lower limb function (Fugl-Meyer Assessment, FMA), balance function (Berg Balance Scale, BBS), and cognitive level (MMSE) of all subjects were evaluated by an experienced physiotherapist before measuring each subject’s biomechanics. The results of the clinical tests are provided in Table 1.

The subjects wore loose-fitting shorts and walking shoes. The obstacle height was set to 15 cm, which is equal to the typical height stairs in the community. A previous study demonstrated that a self-selected walking speed is a good indicator of overall gait performance and is commonly used to assess locomotor ability (Teixeira-Salmela et al., 2008). Therefore, subjects were instructed to walk at a self-selected speed on a 10 m walkway and step over the 15 cm obstacle without contacting it or losing balance. The subjects were instructed to use their affected leg as the leading limb for obstacle crossing (i.e., the first limb to cross the obstacle). Before data collection, the subjects performed five trials at a comfortable speed to familiarize themselves with the experimental environment and action. Data for each subject were then collected from three successive trials. Subjects were instructed to perform the task within their limits of safety and stop if they felt at risk. For security, a therapist walked alongside each subject to assist them, if required. A trial was excluded from analysis if the participant required the therapist’s assistance to maintain balance or tripped over the obstacle.

### Data Processing

Vicon Nexus (Version 1.8.5) and Visual 3D (C-Motion, Inc., United States) were used for kinematic and kinetic data processing. Trajectory data were filtered offline using a dual-pass, 4th-order Butterworth filter with a cutoff frequency of 6 Hz (MacLellan et al., 2015). From these data, a 15-segment biomechanical model (head, trunk, pelvic, 2 forearms, 2 upper arms, 2 hands, 2 thighs, 2 shanks, and 2 feet) was created, based on Visual 3D software. A crossing cycle was defined as the period beginning with the unaffected limb’s heel contact before the obstacle to its next heel contact after crossing the obstacle (Chen et al., 2019). The crossing cycle was divided into four phases: a pre-obstacle double-support phase, an affected-limb swing phase, a middle-crossing double-support phase, and an unaffected-limb swing phase.

Spatiotemporal parameters provide information about step adjustment strategies for successful obstacle crossing and balance. The obstacle crossing was divided into pre-obstacle and post-obstacle phases according to the relative distance between the COM and the marker on the obstacle in AP direction. Step length was measured from heel to heel in the AP direction, and step width was measured from heel to heel in the ML direction during the pre-obstacle and post-obstacle phases. The step-to-step length variability was calculated for each step number during the pre-obstacle and post-obstacle phases (Eq. 1) (Laessoe and Voigt, 2013). Affected and unaffected swing times were calculated from the toe-off to the ipsilateral heel-contact (vertical ground reaction force ≥ 10 N). Two double-support phases were measured: the first from the heel contact of the unaffected limb heel to the toe-off of the affected limb during the pre-obstacle phase (DST1) and the second from the heel contact of the affected limb to the toe-off of the unaffected limb during middle-crossing (DST2). In addition, we examined three distances between the lower limb and the obstacle. The toe-obstacle distance is the horizontal distance between the unaffected-toe marker and the obstacle during the pre-obstacle phase; the heel-obstacle distance (HOD) is the horizontal distance between the heel marker of the affected limb and the obstacle during the post-obstacle phase; the toe-obstacle clearance is the vertical distance between the toe of the swing limb and the obstacle when the toe marker was above the obstacle.

(1) $$S\,t\,e\,p\,l\,e\,n\,g\,t\,h\,v\,a\,r\,i\,a\,b\,i\,l\,i\,t\,y=\frac{S\,t\,e\,p_{p\,o\,s\,t}-S\,t\,e\,p_{p\,r\,e}}{S\,t\,e\,p_{p\,r\,e}}\times 100\%$$

**FIGURE 1:**
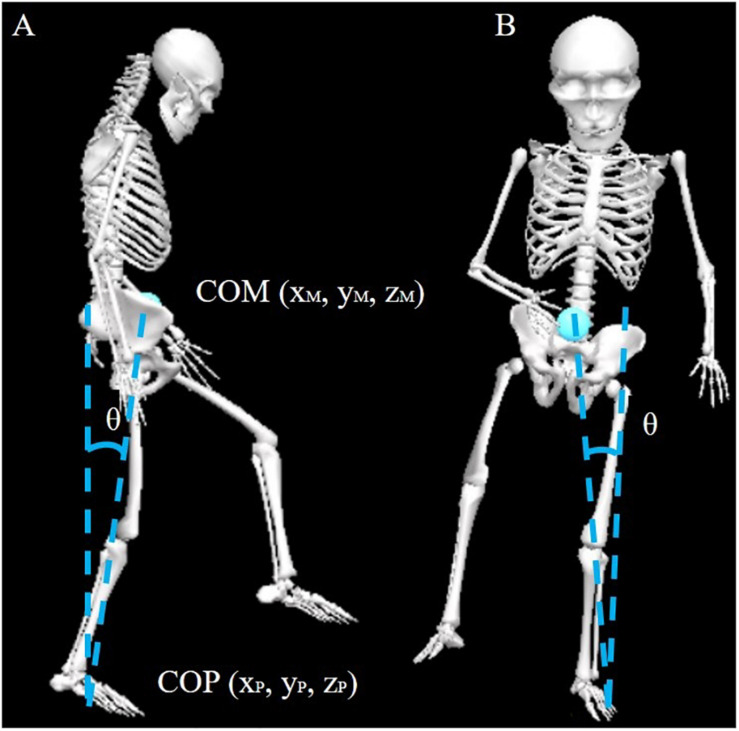
Diagram of COM-COP inclination angles in the sagittal **(A)** and frontal planes **(B)**. COM, center of mass; COP, center of pressure.

The kinematics of the lower limb joint, pelvic, and trunk were calculated to examine the compensatory strategies for successful obstacle crossing. Because the affected side differed among subjects, we defined the direction of tilt and rotation of the contralateral side as [+] and the ipsilateral side as [−] to understand the compensatory strategy better. COM position data were calculated as the weighted sum of each body segment using the whole-body model. The COP is derived from data collected from the two force plates. The averaged AP COM velocity was calculated during the pre-obstacle phase. Instantaneous COM AP and ML velocity were examined when the swing limb toe marker was above the obstacle (i.e., the affected limb, COMV1; the unaffected limb, COMV2). The moment was determined by the smallest distance between the toe marker and the obstacle in AP direction. A previous study demonstrated that the instantaneous COM-COP inclination angles provided information about balance control and fall risk (Figure 1) (Chen and Chou, 2010). Therefore, we calculated instantaneous COM-COP AP and ML inclination angles during the swing phases of the affected and unaffected limbs.

The joint work and joint work contributions provides insight into how to elevate lower limb to step an obstacle (Teixeira-Salmela et al., 2008). Therefore, the joint work (*W*), total work of limb [*W*_*total*_, Eq. (2)], and contributions to the total work [*Cont*_*hip*_, Eq. (3); *Cont*_*knee*_, Eq. (4)] were calculated during the affected- and unaffected-limb swing phases in this study. The biomechanical outcomes from the three trials for each subject were then averaged for subsequent statistical analysis.

(2) $$W_{t\,o\,t\,a\,l}=\left|W_{a\,n\,k\,l\,e}\right|+\left|W_{k\,n\,e\,e}\right|+\left|W_{h\,i\,p}\right|$$

(3) $$C\,o\,n\,t_{h\,i\,p}=\frac{\left|W_{h\,i\,p}\right|}{\left|W_{t\,o\,t\,a\,l}\right|}$$

(4) $$C\,o\,n\,t_{k\,n\,e\,e}=\frac{\left|W_{k\,n\,e\,e}\right|}{\left|W_{t\,o\,t\,a\,l}\right|}$$

### Statistical Analysis

All statistical analyses were performed using IBM SPSS, version 20.0 (SPSS Inc., Chicago, IL, United States). All the calculated variables for both groups were first subjected to a Shapiro–Wilk test. Some variables did not show a normal distribution, and the Mann–Whitney *U* test was applied to test the group-differences. Other continuous variables showing a normal distribution were tested using one-way ANOVA, and the chi-square test was used for categorical variables. The significance level was set at 0.05.

## Results

**FIGURE 2:**
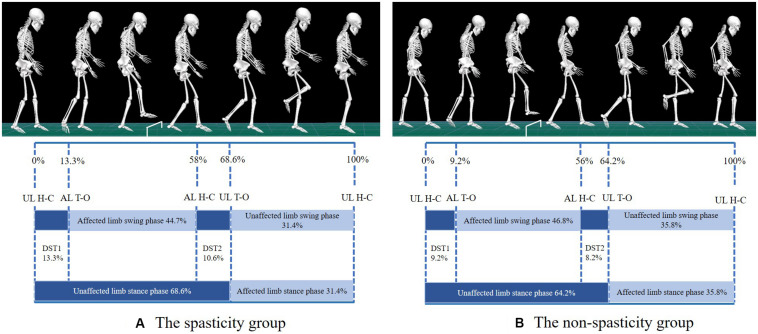
Schematic diagram of typical trials in spasticity group **(A)** and non-spasticity group **(B)** during obstacle crossing. UL, unaffected limb; AL, affected limb; H-C, heel contact; T-O, toe-off; DST1, time between unaffected-limb heel contact and affected-limb toe-off during the pre-obstacle phase; DST2, time between affected-limb heel contact and unaffected-limb toe-off during middle crossing.

Except for MAS, no significant differences were observed in the basic characteristics and clinical test results between the two groups (Table 1). A schematic diagram of typical trials in the spasticity group and the non-spasticity group during obstacle crossing was shown in Figure 2. Additionally, none of the participants exhibited contracture at any of the joints of the lower-limb during the biomechanical test.

### Spatiotemporal Parameters During Obstacle Crossing

Spatiotemporal parameters during obstacle crossing for the two groups were presented in Table 2. Compared to the non-spasticity group, the spasticity group exhibits significantly slower COM AP velocity, slower COMV1_*AP*_, slower COMV2_*AP*_, shorter step length, longer DST1 and smaller toe-obstacle distance during the pre-obstacle phase. An increased step width and a decreased step length were observed in the spasticity group relative to the non-spasticity group during the post-obstacle phase. However, there were no significant differences between the two groups for other spatiotemporal parameters during obstacle crossing.

**TABLE 2:** Comparison of spatiotemporal parameters by study group.

Parameters	Spasticity group	Non-spasticity group	*F*/χ^2^	*P*
COM AP velocity (m/s)**	0.38 (0.32–0.40)	0.58 (0.41–0.77)	12.816	0.002
COMV1_AP_ (m/s)**	0.25 (0.19–0.32)	0.47 (0.34–0.60)	12.542	0.002
COMV1_ML_ (m/s)	0.022 (−0.01–0.025)	0.019 (0.02–0.025)	0.082	0.778
COMV2_AP_ (m/s)**	0.35 (0.27–0.42)	0.53 (0.41–0.64)	10.006	0.005
COMV2_ML_ (m/s)	0.041 (0.01–0.05)	0.036 (0.02–0.05)	0.098	0.758
SL_PRE_ (m)**	0.26 (0.23–0.30)	0.43 (0.37–0.47)	35.100	0.000
Cross step length (m)**	0.43 (0.39–0.47)	0.56 (0.51–0.61)	22.075	0.000
SL_POST_ (m)*	0.28 (0.21–0.34)	0.45 (0.36–0.54)	13.666	0.002
SW_PRE_ (m)	0.10 (0.07–0.13)	0.08 (0.03–0.13)	0.608	0.446
SW_POST_ (m)*	0.15 (0.13–0.18)	0.11 (0.08–0.15)	4.533	0.047
DST1 (%)*	13.34 (10.08–16.59)	9.23 (7.45–11.02)	5.381	0.032
DST2 (%)	10.60 (7.68–13.52)	8.18 (6.26–10.10)	1.031	0.157
Affected swing time (%)	44.67 (41.41–47.93)	46.79 (43.43–50.15)	2.180	0.323
Unaffected swing time (%)	31.40 (0.26–0.36)	35.80 (0.30–0.42)	1.681	0.211
TOD (m)**	0.12 (0.10–0.15)	0.22 (0.16–0.28)	13.456	0.002
TOC (m)	0.11 (0.06–0.17)	0.13 (0.09–0.16)	0.211	0.651
HOD (m)	0.07 (0.03–0.10)	0.10 (0.06–0.14)	1.905	0.184

COM AP velocity, anterior–posterior velocity of COM during the pre-obstacle phase; COMV1_*AP*_, anterior–posterior velocity of COM when the affected-limb toe marker is above the obstacle; COMV1_*ML*_, medio-lateral velocity of COM when the affected-limb toe marker is above the obstacle; COMV2_*AP*_, anterior–posterior velocity of COM when the unaffected-limb toe marker is above the obstacle; COMV2_*ML*_, medio-lateral velocity of COM when the unaffected-limb toe marker is above the obstacle; SL, step length; SW, step width; DST1, time between unaffected-limb heel contact and affected-limb toe-off during the pre-obstacle phase; DST2, time between affected-limb heel contact and unaffected-limb toe-off during obstacle crossing; TOD, horizontal distance between the unaffected-limb toe marker and the obstacle during the pre-obstacle phase; HOD, horizontal distance between the affected-limb heel marker and the obstacle during the post-obstacle phase; TOC, vertical distance between the toe of the swing limb and the obstacle when the toe marker is above the obstacle. *P < 0.05, **P < 0.01.

**FIGURE 3:**
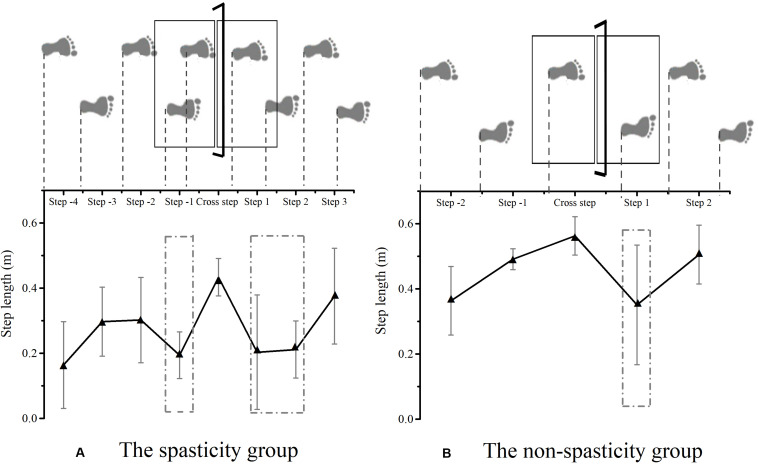
Schematic explanation of step adjustments strategy among the spasticity group **(A)** and the non-spasticity group **(B)** during obstacle crossing. **(A)** The spasticity group; **(B)** the non-spasticity group; Mean values and standard deviation for each step in the two groups were plotted as black and gray lines, respectively. The dash-dotted rectangular area represents the location of step adjustment.

Figure 3 shows the step adjustment among the two groups during obstacle crossing. Compared to the non-spasticity group, an additional shortened step was observed in the spasticity group during the pre-obstacle phase, in front of the obstacle, prior to the actual crossing maneuver. Between steps −2 and −1, a negative and positive change percentage of the step length was observed in the spasticity group (−38.32 percentage points; 95% CI, 20.44. 56.20) and the non-spasticity group (48.56 percentage points; 95% CI, 9.02. 88.10), respectively. During the post-obstacle phase, an additional shortened step was also observed in the spasticity group prior to restoring step length between steps 1 and 3. Between cross step and step 1, a negative change in the percentage of the step length was found in the spasticity group (−53.98 percentage points; 95% CI, 28.61. 79.35) and the non-spasticity group (−39.37 percentage points; 95% CI, 15.98. 62.76), respectively. However, no significant difference was observed between the two groups (*F* = 0.877, *P* = 0.361). Additionally, a positive change in the percentage of the step length was found in the spasticity group between steps 2 and 3 (106.06 percentage points; 95% CI, 13.82. 198.29), and a positive change in the percentage of the step length was found in the non-spasticity group between steps 1 and 2 (91.79% percentage points; 95% CI, 9.28. 174.3). No significant group-differences were found (*F* = 0.065, *P* = 0.802).

### Kinematics of Trunk, Pelvic, Lower Limb Joint, and COM-COP Inclination Angle During Swing Phases

**FIGURE 4:**
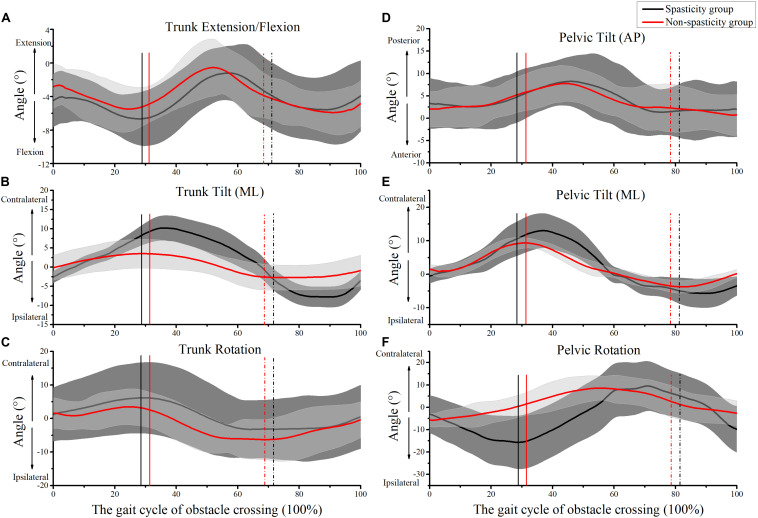
Kinematics of trunk and pelvic during obstacle crossing. **(A)** Trunk extension/flexion; **(B)** trunk medio-lateral (ML) tilt; **(C)** trunk rotation; **(D)** pelvic anterior–posterior (AP) tilt; **(E)** pelvic ML tilt; **(F)** pelvic rotation. The mean times that the affected-limb was above the obstacle in the spasticity group (29.12 ± 4.44%) and the non-spasticity group (30.38 ± 2.48%) were plotted as black and red vertical lines, respectively. The mean times that the unaffected-limb toe was above the obstacle in the spasticity group (81.61 ± 5.62%) and the non-spasticity group (78.68 ± 5.29%) were plotted as black and red vertical dash-dot lines, respectively.

Figure 4 shows the kinematics of the trunk and pelvic during obstacle crossing. Compared to the non-spasticity group, the trunk lateral tilt, pelvic lateral tilt, and pelvic rotation angles were higher in the spasticity group when the affected limb toe was above the obstacle (Table 3A). The trunk lateral tilt angle was higher in the spasticity group relative to the non-spasticity group when the unaffected-limb toe was above the obstacle (Table 3B).

**TABLE 3:** Mean joint angle (95% confidence intervals) of the swing limb when the affected-limb toe or unaffected-limb toe was above the obstacle.

	(A) Affected limb swing				(B) Unaffected limb swing			
Crossing angle (°)	Spasticity group	Non-spasticity group	*F*	*P*	Spasticity group	Non-spasticity group	*F*	*P*
Trunk flexion (−)/extension (+)	−6.45 (−8.95–−3.95)	−5.19 (−7.10–−3.27)	0.756	0.396	−5.12 (−8.05–−2.18)	−6.00 (−7.84–−4.17)	0.296	0.593
Trunk lateral tilt Contra (+)/Ipsi (−) **^,#^	8.72 (6.43–11.01)	3.63 (0.55–6.71)	9.464	0.007	−5.08 (−6.70–−3.46)	−1.86 (−1.97–−2.06)	8.115	0.011
Trunk rotation Contra (+)/Ipsi (−)	5.10 (3.60–6.59)	3.03 (0.24–5.83)	2.448	0.135	−2.64 (−6.87–1.58)	−4.64 (−8.83–−0.46)	−0.266	0.790
Pelvic tilt anterior (−)/posterior (+)	5.54 (2.49–8.59)	5.77 (2.57–8.96)	0.013	0.912	1.61 (−3.19–6.41)	2.37 (−1.78–6.54)	0.071	0.793
Pelvic lateral tilt Contra (+)/Ipsi (−)*	10.77 (9.14–12.40)	7.98 (6.32–9.62)	7.264	0.015	−4.65 (−9.32–0.02)	−1.68 (−4.53–1.16)	1.322	0.265
Pelvic rotation Contra (+)/Ipsi (−)**	−15.56 (−19.47–−11.65)	−0.79 (−5.13–−3.55)	32.717	0.000	6.59 (5.06–8.13)	4.42 (0.63–9.46)	1.070	0.315
Hip flexion (+)/extension (−)	37.50 (31.17–43.83)	43.12 (35.48–50.53)	1.632	0.218	27.41 (19.17–35.65)	26.38 (16.81–35.94)	0.035	0.855
Hip abduction (+)/adduction (−)	9.29 (5.46–13.13)	6.16 (2.36–9.95)	−1.709	0.087	8.64 (2.27–15.01)	10.67 (3.59–17.74)	0.233	0.635
Hip rotation internal (+)/external (−)*	7.95 (7.48–13.16)	17.33 (10.07–26.81)	−2.165	0.030	12.16 (7.77–16.56)	17.29 (11.48–23.10)	2.646	0.121
Knee flexion (−)/extension (+)*	−58.84 (−68.98–−48.69)	−74.61 (−87.81– −61.42)	4.780	0.042	−93.81 (−102.62–−85.02)	−96.48 (−105.99–−86.97)	0.215	0.649
Ankle DF (+)/PF (−)*	5.18 (0.44–9.90)	11.72 (7.19–16.24)	4.937	0.039	14.04 (8.18–19.88)	17.73 (13.09–22.35)	1.158	0.296

DF, dorsiflexion; PF, plantarflexion; Contra, contralateral; Ipsi, ipsilateral; *reflects a significant difference between the two groups when the affected-limb toe was above the obstacle (P < 0.05); **reflects P < 0.01; ^#^reflects a significant difference between the two groups when the unaffected-limb toe was above the obstacle (P < 0.05).

**FIGURE 5:**
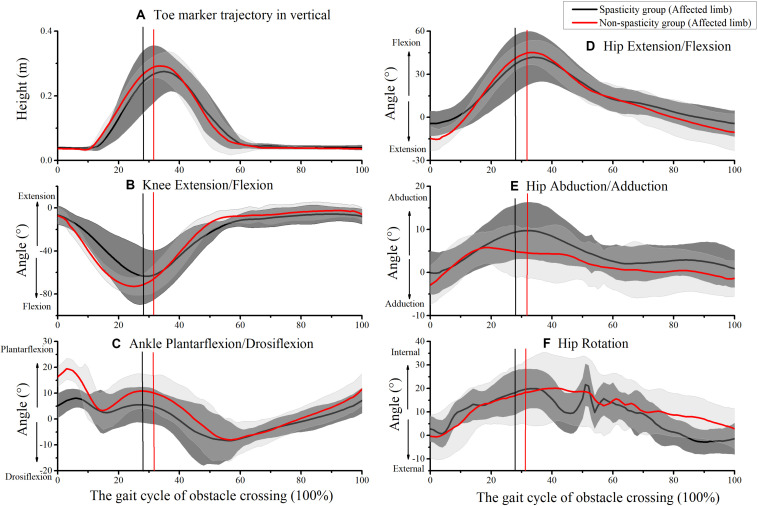
Kinematics of affected-limb joints during obstacle crossing. **(A)** Toe marker vertical trajectory; **(B)** knee extension/flexion; **(C)** ankle plantarflexion/dorsiflexion; **(D)** hip extension/flexion; **(E)** hip abduction/adduction; **(F)** hip rotation. The mean times that the affected-limb toe was above the obstacle in the spasticity group (29.12 ± 4.44%) and the non-spasticity group (30.38 ± 2.48%) were plotted as black and red vertical lines, respectively.

Figure 5 shows the kinematics of the affected-limb hip, knee, and ankle during obstacle crossing. During the affected-limb swing phase, the hip rotation, knee flexion, and ankle dorsiflexion values were lower in the spasticity group relative to the non-spasticity group when the affected-limb toe was above the obstacle (Table 3A). No significant difference was observed between the two groups for toe-obstacle clearance (Table 2).

**FIGURE 6:**
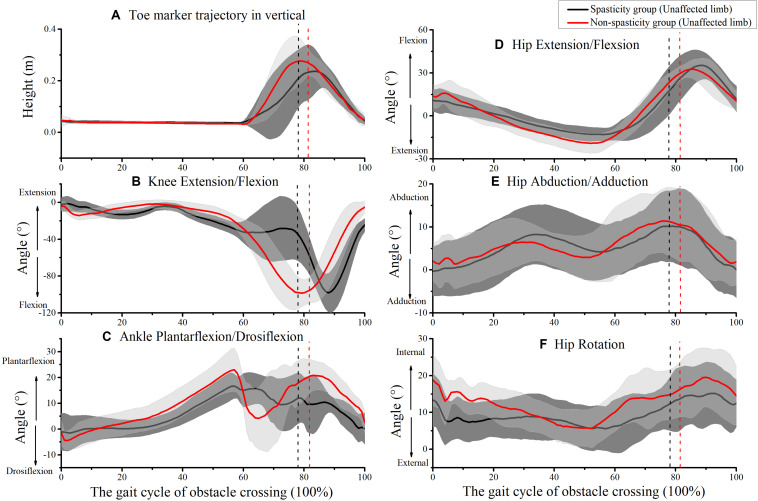
Kinematics of unaffected-limb joints during obstacle crossing. **(A)** Toe marker vertical trajectory; **(B)** knee extension/flexion; **(C)** ankle plantarflexion/dorsiflexion; **(D)** hip extension/flexion; **(E)** hip abduction/adduction; **(F)** hip rotation. The mean times that the unaffected-limb toe was above the obstacle in the spasticity group (81.61 ± 5.62%) and the non-spasticity group (78.68 ± 5.29%) were plotted as black and red vertical dash-dot lines, respectively.

Figure 6 shows the kinematics of the unaffected-limb hip, knee, and ankle during obstacle crossing. However, no significant differences were observed between the two groups for all kinematic parameters during the unaffected-limb swing phase (Tables 2, 3).

**FIGURE 7:**
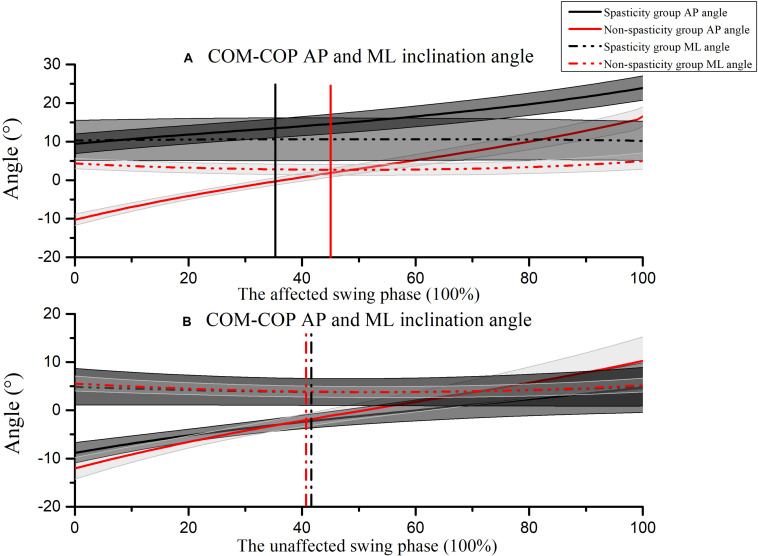
COM-COP inclination angles during **(A)** the affected-limb swing phase, and **(B)** the unaffected-limb swing phase. Positive values indicate that the COM is in front of the COP. Toe-off and heel contact are indicated as 0 and 100%, respectively. The mean time that the affected-limb toe was above the obstacle in the spasticity group (35.47 ± 7.49%) and the non-spasticity group (45.41 ± 3.53%) were plotted as black and red vertical lines, respectively. The mean time that the unaffected-limb toe was above the obstacle in the spasticity group (41.08 ± 8.09%) and the non-spasticity group (40.96 ± 4.71%) were plotted as black and red vertical dash-dot lines, respectively.

Figure 7 shows the COM-COP AP and ML inclination angles during the affected-limb swing phase and the unaffected-limb swing phase. During the swing phase of the affected limb, the COM-COP AP inclination angle was larger in the spasticity group (14.95°; 95% CI, 13.12. 16.77) than that in the non-spasticity group (2.45°; 95% CI, 1.53. 3.37) when the affected-limb toe marker was above the obstacle (*F* = 772.378, *P* = 0.000). Furthermore, the COM-COP ML inclination angle was larger in the spasticity group (9.29°; 95% CI, 4.08. 14.50) than that in the non-spasticity group (3.57°; 95% CI, 2.69. 4.46) when the affected-limb toe marker was above the obstacle (*F* = 4.754, *P* = 0.043). No significant differences were observed in the COM-COP AP and ML inclination angles between the two groups during the unaffected-limb swing phase.

### Work and Work Contributions of Joints During Swing Phases

Table 4 shows the joints work and work contributions of the swing limb during the affected-limb and the unaffected-limb swing phases. During the swing phase of the affected-limb, the lower knee work, lower knee work contributions and higher hip work contributions were observed in the spasticity group than in the non-spasticity group. However, no significant differences were observed in work and work contributions between the two groups during the unaffected-limb swing phase.

**TABLE 4:** Mean joint work (95% confidence intervals) of the swing limb during the affected-limb swing phase **(A)** or unaffected-limb swing phase **(B)**.

Joint work and work contributions	(A) Affected limb swing				(B) Unaffected limb swing			
	Spasticity group	Non-spasticity group	*F*	*P*	Spasticity group	Non-spasticity group	*F*	*P*
Ankle work (J)	0.13 (0.05–0.21)	0.19 (0.11–0.27)	1.329	0.264	0.49 (0.37–0.62)	0.61 (0.50–0.72)	2.636	0.124
Knee work (J)*	1.06 (0.64–1.48)	2.08 (1.05–3.11)	5.072	0.037	28.71 (23.45–33.95)	30.41 (22.55–38.27)	0.188	0.670
Hip work (J)	2.98 (1.94–4.02)	2.75 (1.71–3.79)	0.122	0.731	19.19 (11.97–26.41)	20.04 (12.09–27.98)	0.033	0.859
Limb total work (J)	4.18 (2.79–5.56)	5.02 (3.39–6.65)	0.811	0.380	48.39 (36.31–60.47)	51.05 (36.06–66.07)	0.105	0.750
Hip work contributions (%)**	71.05 (65.56–76.54)	55.77 (46.14–65.40)	10.820	0.004	38.02 (33.05–43.00)	38.37 (32.50–44.24)	0.011	0.918
Knee work contributions (%)**	25.16 (19.60–30.71)	33.43 (23.93–42.93)	8.815	0.008	60.92 (56.07–65.76)	60.29 (54.72–65.87)	0.038	0.848

*Reflects a significant difference between the two groups during the affected-limb swing phase (P < 0.05). **Reflects *P* < 0.01.

## Discussion

The purpose of this study was to systematically examine the step adjustment and compensatory strategies used by stroke survivors with knee extensor spasticity during obstacle crossing. In the present study, we compared kinematics and kinetics of a spasticity group versus a non-spasticity group during the crossing of a 15 cm obstacle and identified knee extensor spasticity-related differences in step adjustment and compensatory strategies. Our results demonstrate that stroke survivors with knee spasticity use different step adjustment and compensatory strategies during the different phases of the obstacle crossing. As expected, the coupling of movement between the pelvic and trunk is an important compensatory strategy for successful obstacle crossing, but it sacrifices some balance in the sideways direction (Rhee and Kim, 2015; van Vugt et al., 2019). These results revealed the step adjustment and compensatory strategies for obstacle crossing and also provide insight into the design of rehabilitation interventions for fall prevention in stroke survivors with knee extensor spasticity.

When the stroke survivors approach the obstacle, the steps must be modified in due time to minimize disturbance to the gait, even in the absence of temporal constraints (Nakano et al., 2014). Step adjustment strategies are necessary to cross the obstacle successfully and maintain balance during complex community ambulation (Laessoe and Voigt, 2013). Several studies reported that the step length and COM AP velocity were significantly smaller in stroke survivors than in healthy controls (healthy controls reference value, 0.69 m, 1.05 m/s) (Said et al., 2008). Therefore, we expected a shorter step length and slower COM AP velocity in the spasticity group than the non-spasticity group, due to the knee extensor spasticity. As expected, a shorter step length and slower COM AP velocity were observed in the spasticity group relative to the non-spasticity group during the pre-obstacle phase in the present study. This finding was consistent with comparisons between stroke and healthy subjects in previous studies (Said et al., 2008; Nakano et al., 2014; Chen et al., 2019). Nakano et al. (2014) suggested that the short-step strategy was used by stroke survivors to step over the obstacle, which probably intended to enhance the accuracy of swing and maintain stability. The smaller step length positions the COP closer to the COM, which could result in smaller moment arms for bodyweight of the stance limb, and requires less muscular effort to maintain balance (Chou et al., 2003; Said et al., 2008). In addition, the slower COM AP velocity has the potential advantage of easily regaining stability (Said et al., 2008). More importantly, the spasticity group added an additional shortened step between step −2 and cross step prior to the actual crossing maneuver. Successful community ambulation depends on the ability to adapt gait to the environment and to diverse behavioral goals (Malone et al., 2016). A possible explanation for this finding might be that the spasticity group integrates the available sensory and environmental information to initiate an appropriate or cautious movement strategy, based on their functional level and knee extensor spasticity.

Den Otter et al. (2005) reported that stroke survivors generally preferred a lengthening of the step to cross the obstacle under a time constraint. They concluded that the ability to adequately modify the stepping pattern in response to imposed spatiotemporal constraints is impaired in patients with stroke, especially when modifications must be performed under time pressure. A previous study reported that stroke survivors adopted a short-step strategy to step over the obstacle in the absence of time constraints (Nakano et al., 2014). However, the non-spasticity group lengthened their steps during pre-obstacle in the present study without time constraints. The potential explanation for the step lengthening is that the crossing limb will be in a leading position and that visual information on the crossing limb will be available continuously. In contrast, the shortened step will generally result in a situation in which the crossing limb will be in a trailing position, so that visual information will not, or will only partially, be available. This may make a lengthened step easier to implement, especially for stroke survivors who are more dependent on visual information (Den Otter et al., 2005; Laessoe and Voigt, 2013; Watanabe et al., 2016). The differences in the basic characteristics of subjects may be being the reason why the present study is inconsistent with Nakano et al. (2014). In the present study, the sample was composed of mild compromised post-stroke individuals. Additionally, the present findings on long-step strategy are in line with results obtained in healthy elderly (Den Otter et al., 2005). Therefore, the step adjustment strategy may be related to the functional level of the subjects.

Our analysis of the toe-obstacle distance, cross step length and heel-obstacle distance data supports the proposal that the spasticity group utilized the above-mentioned short-step strategy for successful obstacle crossing. A previous study suggested that the short-step strategy was used to approach the obstacle in stroke survivors, which probably intended to enhance the accuracy of swing and maintain stability (Nakano et al., 2014). Therefore, we expected that the shorter toe-obstacle distance in the spasticity group than in the non-spasticity group (healthy controls reference value, 0.20 m) (MacLellan et al., 2015). The spasticity disrupts the pattern of agonist-antagonist activation and alters the net effect of the forces generated by muscle groups, as confirmed by a previous study (Singer et al., 2013). We further expected the smaller cross step length and heel-obstacle distance in the spasticity group compared to the non-spasticity group (healthy controls reference value, 0.78 and 0.22 m) (Said et al., 2008; Chen et al., 2019). In the present study, our results showed that the toe-obstacle distance was shorter in the spasticity group, while no group-differences were observed in cross step length and heel-obstacle distance between the two groups. The usage the short-step strategy by the spasticity group may provide two advantages for obstacle crossing (Nakano et al., 2014). One is foot placement accuracy, the other is safety. A shorter toe-obstacle distance may be the result of the short-step adjustment strategy, to shorten the distance between the swing limb and the effective swing-foot position. Furthermore, the slower COM AP velocity may provide time to modify the swing-foot position and easily regain stability (Said et al., 2008; Mizusawa et al., 2017). Although a shorter toe-obstacle distance provides an advantage in successfully crossing obstacles, it may also reduce the space of the swing limb and increase the risk of tripping over the obstacle. A previous study reported that the shorter heel-obstacle distance in stroke survivors compared with healthy controls might place stroke survivors at risk of actual contact with or tripping over the obstacle (Said et al., 2005; Chen et al., 2019). However, no significant difference was found in the cross step length and heel-obstacle distance between the spasticity and the non-spasticity groups in the present study. A potential explanation is that the spasticity group used the short-step strategy during the pre-obstacle phase to obtain a similar cross step length as the non-spasticity group.

Stroke survivors may face difficulty in muscle recruitment and knee motion range, and knee extensor spasticity may further exacerbate this negative effect (Singer et al., 2013). Therefore, we expected the lower knee flexion angle and knee work contributions occur in the spasticity group compared to non-spasticity group (healthy controls reference value, 97.46°) (Chen et al., 2019). Due to a lack of knee flexion, a “hip abduction strategy” is typically used by stroke survivors for limb elevation during obstacle crossing (Lu et al., 2010; Chen et al., 2019). We further expected greater hip abduction angle and hip work contributions in the spasticity group compared to the non-spasticity group (healthy controls reference value, 0.14°) (Chen et al., 2019). As expected, the spasticity group exhibited lower knee flexion when the affected-limb toe was above the obstacle. However, no significant difference was found in hip abduction between the two groups when the affected-limb toe was above the obstacle. This finding is inconsistent with the results of a previous study (Chen et al., 2019). A possible explanation for this inconsistency might be that in the present study the hip kinematics were calculated relative to the pelvic, and the angle of pelvic tilt in the frontal plane was higher in the spasticity group than the non-spasticity group in our study. This may lead to underestimation of the true hip abduction in the global coordinate system. Therefore, we calculated the relative ML distance between the ankle and ipsilateral hip joint when the affected-limb toe was above the obstacle. Our results showed the higher ML distance between the ankle and ipsilateral hip joint in the spasticity group (0.102 m; 95% CI, 0.061. 0.145) than the non-spasticity group (0.048 m; 95% CI, 0.025. 0.071) when the affected-limb toe was above the obstacle (*F* = 5.56, *P* = 0.030). Additionally, the results showed that hip work contributions were higher and knee work contributions were lower in the spasticity group than in the non-spasticity group. These results indicate that the hip may be a key component of limb elevation.

The coupling of movement between the pelvic and trunk contributed to the compensatory strategy for complex tasks. Han et al. (2017) and Chen et al. (2019) reported that the pelvic and trunk lateral tilt angles were larger in stroke survivors than in healthy controls (healthy controls reference value, 4.5°). Our results suggest that the coupling of movement between the pelvic and the trunk is an important compensatory strategy in the spasticity group during obstacle crossing. As we hypothesized, the pelvic and trunk lateral tilt angles in the spasticity group were indeed larger than those in the non-spasticity group. This finding was similar to that of a previous study (Han et al., 2017; Chen et al., 2019; van Vugt et al., 2019). In the current study, the spasticity group initially shifted their weight to the unaffected side, then laterally tilted the pelvic and trunk toward the unaffected side and abducted the hip to elevate the swing foot. By adopting this proximal movement compensatory strategy, the spasticity group was able to cross the obstacle successfully.

Chen and Chou (2010) suggested that the COM-COP inclination angle could sensitively identify individuals with imbalance and fall risk among stroke survivors during walking and obstacle crossing. A previous study reported that after using the pelvic lateral tilt strategy, stroke survivors showed greater instability in the ML direction during the push-off and landing phases (Chen et al., 2019). Therefore, we expected the COM-COP ML inclination angle would be larger in the spasticity group than in the non-spasticity group (healthy controls reference value, 4.09°) (Lee and Chou, 2006). As expected, we observed that the COM-COP ML inclination angle was larger in the spasticity group than in the non-spasticity group when the affected-limb toe was above the obstacle. In addition, the COM-COP AP inclination angle was larger in the spasticity group when the affected-limb toe was above the obstacle. Although a proximal movement compensatory strategy may improve obstacle crossing, it sacrifices some balance in the sideways direction. Even though the relation between COM-COP ML can be related to balance disruption, this can also be a compensatory strategy to complete the task. Similar results were found in posture control during gait in adults with hereditary spastic paraparesis (van Vugt et al., 2019). The adults with hereditary spastic paraparesis had slower walking velocity, more time spent in double stance, larger step widths, and greater trunk lateral tilt than the healthy controls. These results suggest that the individuals with hereditary spastic paraparesis adjust their gait to minimize the instability arising from their impairments but have residual deficits in ML stability.

Spatiotemporal parameters measured after obstacle crossing provided information about the reestablish of a walking pattern and balance control. Said et al. (2008) demonstrated that a reduction in step length after the obstacle could represent a reestablishment of the gait pattern. Therefore, we expected a smaller step length in the spasticity group than in the non-spasticity group (healthy controls reference value, 0.69 m) (Said et al., 2008). As expected, our results show that the step length was indeed smaller in the spasticity group than in the non-spasticity group. Interestingly, an additional shortened step was observed prior to restoring step length between step 1 and step 3 in the spasticity group. Furthermore, we observed that the step width in the spasticity group was longer than that in the non-spasticity group. This indicates that a short-step and increase step width strategy was adopted to reestablish the walking pattern and balance control because the affected limb is less capable of providing single support.

In the present study, the sample size was calculated using parametric methods before the biomechanics test while some measures were tested using non-parametric methods. In our results, the heel-obstacle distance, step width in pre-obstacle and trunk rotation angle were not showed normal distributions, and using Mann–Whitney *U* test to test the group-differences. However, no significant differences was found between the two groups. The central limit theorem essentially demonstrated that even though the distribution of individual observations is not normal, with an increasing sample size, the distribution of a mean becomes more normal (Divine et al., 2013). Additionally, the number of subjects actually recruited (*n* = 20) is greater than the result of the sample size calculations (*n* = 14). Therefore, this discrepancy may be not affecting the results and conclusions of the present study.

Experimental studies on the step adjustment and compensatory strategies for obstacle crossing in stroke survivors with knee extensor spasticity may help in the development of better fall preventive training programs. In the present study, our results demonstrate that the trunk and pelvic movement compensatory strategies may improve obstacle crossing, but it sacrifices some balance in the sideways direction. A previous study suggested that the degree of trunk movement was restricted to enable body stability during the early stage of motor learning and balance development (Rhee and Kim, 2015). When trunk control must respond to external disturbances or to compensatory movement of other joints, the risk of falling may be increased. This indicates that trunk stabilization exercises or core muscle strength enhancement in early stage of rehabilitation may play an important role in fall prevention for stroke survivors (Shin and Don Kim, 2016; Lee et al., 2018). Additionally, recent clinical studies found that trunk stabilization exercises influence the muscle tone of the distal part, which can be explained as a decrease in spasticity because the excitation of the spinal cord motor neurons decreased after the trunk stabilization exercise (Rhee and Kim, 2015). Furthermore, in the present study, in the absence of time pressure, stroke survivors with knee extensor spasticity preferred short-step adjustment strategies, which is differed than the non-spasticity group. A previous study demonstrated that the ability to adequately “on-line” modify the stepping pattern in response to imposed spatiotemporal constraints is impaired for stroke and older adults, especially when modifications must be performed under time pressure (Den Otter et al., 2005). Therefore, trunk stabilization exercises and repeated obstacle avoidance exercises under time constraint may be an effective intervention to prevent falls (Chien and Hsu, 2018).

This study has several limitations. First, considering the insufficient strength and spasticity of the affected limb during the stance phase, we did not instruct the subjects to step over the obstacle with their unaffected side due to safety issues. Therefore, we collected data only when the affected limb was the leading limb during obstacle crossing, but this does not influence the results and conclusions of this study. Second, the obstacle height was set at 15 cm, which is equal to the height between stairs in the community. We did not assess the biomechanics of crossing obstacles of different heights (e.g., the percentage of leg length). A previous study demonstrated that stair climbing is a critical factor for restoring independent daily living to stroke survivors (Morone et al., 2018). Although obstacle crossing is not identical to stair climbing, the compensatory strategies of affected-limb elevation during obstacle crossing are still applicable to limb elevation during stair climbing because the height of the obstacle is the same. Third, the spasticity of the other lower limb muscles (e.g., hip adductors, ankle dorsiflexors, and ankle plantar flexors) was not assessed in the present study. In future work, this should be combined with EMG analysis to investigate compensatory strategies used by stroke survivors with single and multiple spasticity muscle groups, which has important theoretical value in rehabilitation treatment and fall prevention for stroke survivors with spasticity.

## Conclusion

During the pre-obstacle phase, stroke survivors with knee extensor spasticity adopted a short-step strategy to approach the obstacle, while stroke survivors without knee extensor spasticity used long-step strategy. During the affected-limb swing phase, the combined movement of the pelvic, and trunk lateral tilt and hip abduction is an important compensatory strategy for successful obstacle crossing, but it sacrifices some balance in the sideways direction. During the post-obstacle phase, short-step and increase step width strategies were adopted to reestablish the walking pattern and balance control. Trunk stabilization exercises and repeated obstacle avoidance exercises under time constraints may be an effective intervention to prevent falls.

## Data Availability Statement

The raw data supporting the conclusions of this article will be made available by the authors, without undue reservation, to any qualified researcher.

## Ethics Statement

The studies involving human participants were reviewed and approved by Ethics Committee of Shanghai Seventh People’s Hospital. The patients/participants provided their written informed consent to participate in this study.

## Author Contributions

S-JH, X-MY, X-BW, and W-XN conceived and designed the study. S-JH and X-MY recruited subjects and collected the basic characteristics of subjects. X-BW performed clinical assessment. S-JH, X-MY, and KW performed the experiments. S-JH, L-JW, and XW made a contribution to data analysis. S-JH and W-XN wrote this manuscript. All authors had read and approved the manuscript.

## Conflict of Interest

The authors declare that the research was conducted in the absence of any commercial or financial relationships that could be construed as a potential conflict of interest.
